# Omega-3 and ranibizumab for age-related macular degeneration: A systematic review protocol

**DOI:** 10.1097/MD.0000000000014516

**Published:** 2019-03-15

**Authors:** Yan Meng, Hong-wei Liu, Peng Sun, Ping-ping Zhou, Jian-jie Wang

**Affiliations:** a Department of Ophthalmology, First Affiliated Hospital of Jiamusi University; b Department of Immunology, Jiamusi University, Jiamusi, China.

**Keywords:** age-related macular degeneration, efficacy, Omega-3, ranibizumab, safety, systematic review

## Abstract

**Background:**

Omega-3 and ranibizumab (O3R) has been reported to treat age-related macular degeneration (ARMD) effectively. However, up to the present, no systematic review specifically addressed the efficacy of O3R for the treatment of ARMD. Therefore, in this study, we will propose to assess the efficacy and safety of O3R for the treatment of ARMD.

**Methods:**

We will search PUMBED, EMBASE, CINAHI, Cumulative Index to Nursing and Allied Health Literature, Allied and Complementary Medicine Database, Cochrane Library, Chinese Biomedical Literature Database, China National Knowledge Infrastructure, VIP Information, Wanfang Data, as well as the gray literature from inception up to the present. We will accept randomized controlled trials for assessing the efficacy and safety of O3R for ARMD. The primary outcomes include change in best corrected visual acuity and central retinal thickness. The secondary outcomes consist of changes in subfoveal choroidal thickness, macular atrophy, retinal average sensitivity, contrast sensitivity, glare disability, and quality of life. In addition, incidence and severity of adverse events will also be evaluated. Cochrane Collaboration tool will be used to assess the risk of bias for each included study. In addition, Grading of Recommendations Assessment, Development, and Evaluation tool will be utilized to assess the overall strength of the evidence. Two authors will independently carry out all procedures and any divergences will be solved through discussion with a third author. If it is possible, we will conduct meta-analysis and subgroup analysis concerning different interventions, risk of bias, and outcome measurements.

**Results:**

In this proposed study, we outline details of the aims and methods of efficacy and safety of O3R for the treatment of ARMD.

**Conclusion:**

The findings of this systematic review will summarize current evidence of O3R for the treatment of patients with ARMD.

**Dissemination and ethics:**

The results of the present study are expected to be published by peer-reviewed journals. This is a literature-based study. Thus, ethical approval is unnecessary for this study.

**Systematic review registration:**

PROSPERO CRD42019121177.

## 1 Introduction

Age-related macular degeneration (ARMD) is one of the most common reasons for severe visual impairment in patients aged 50 years and above.^[1,2]^ Correspondingly, visual function is greatly decreased as the disease progresses.^[3,4]^ Thus, it is generally thought to be the leading cause of blindness in patients with such condition.^[5–8]^ Previously, a US study has reported that >6.5% people older than 40 years have been diagnosed with ARMD.^[9]^ Of them, about 1.75 million patients are in the advanced stage.^[9]^ Most importantly, such number is still expected up to the 2.95 million among the overall population ages.^[10]^

Currently, numerous studies have reported using intravitreal injection of ranibizumab to treat ARMD, and have already achieved promising efficacy.^[11–14]^ However, such treatment still has certain limitations for some patients, including the limited efficacy and severe adverse events.^[15,16]^ In such situation, it would be great if an effective adjunctive therapy with fewer adverse events can be added to ranibizumab for the treatment of ARMD. Fortunately, Omega-3 is also reported to treat ARMD by many clinical trials effectively with fewer adverse events.^[17–24]^ Furthermore, several trials have been conducted to investigate the efficacy and safety of Omega-3 and ranibizumab (O3R) for the treatment of ARMD.^[18,20,24]^ However, no systematic review has been performed to evaluate the efficacy and safety of O3R for the treatment of ARMD. This study will assess the efficacy and safety of O3R for the treatment of ARMD.

## 2 Methods and analysis

### 2.1 Study registration

The protocol of this systematic review has been registered on PROSPERO (CRD42019121177), and has reported following the Preferred Reporting Items for Systematic Reviews and Meta-analysis Protocol.^[25]^

### 2.2 Study selection criteria

#### 2.2.1 Types of studies

We will consider randomized controlled trials (RCTs) of O3R for the treatment of ARMD for inclusion. However, non-RCTs, quasi-RCTs, nonclinical trials, and noncontrol trials will not be considered.

#### 2.2.2 Types of participants

We will accept any diagnosed criteria of ARMD without restrictions of race, sex, and age.

#### 2.2.3 Types of interventions

We will include studies that have implemented O3R alone as an experimental treatment regardless of its treatment form, dosage, frequency, and duration. Control therapy can be any kind of therapy, except the O3R.

#### 2.2.4 Types of outcomes

Studies will be considered for inclusion if they report at least one of the following outcome measurements.

##### 2.2.4.1 Primary outcome

1. Change in best corrected visual acuity.
2. Central retinal thickness.

##### 2.2.4.2 Secondary outcome

1. Change in subfoveal choroidal thickness.
2. Macular atrophy.
3. Retinal average sensitivity.
4. Contrast sensitivity.
5. Glare disability.
6. Quality of life.
7. Incidence and severity of adverse events.

### 2.3 Search strategy for study identification

#### 2.3.1 Electronic databases searches

We will search the following databases for relevant studies from the inception to the present: PUMBED, EMBASE, CINAHI, Cumulative Index to Nursing and Allied Health Literature, Allied and Complementary Medicine Database, Cochrane Library, Chinese Biomedical Literature Database, China National Knowledge Infrastructure, VIP Information, and Wanfang Data. The search strategy of Cochrane Library is detailed in Table 1. Identical search strategies will be used for other electronic databases.

**Table 1:**
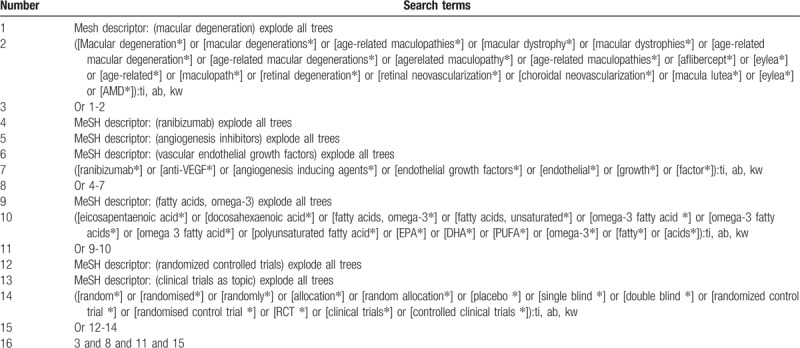
Search strategy applied in Cochrane Library database.

#### 2.3.2 Other resources searches

We will also search resources of gray literature, such as Gray Literature in Europe, National Library of Medicine Bookshelf, Clinical Trials Registry, and Reference lists of relevant reviews and included trials.

### 2.4 Study selection

**Figure 1:**
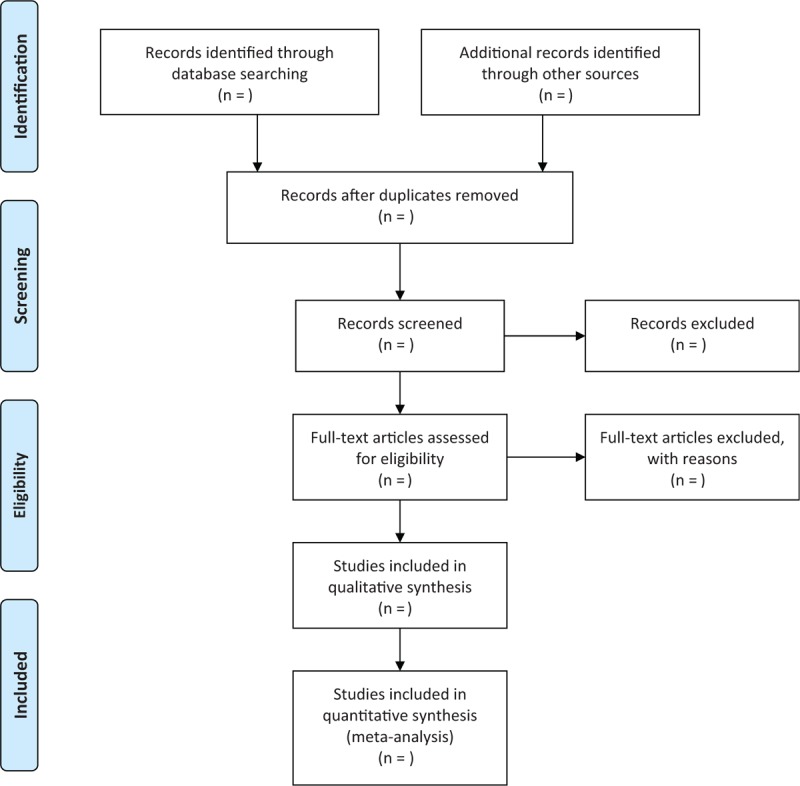
Process of study selection.

Two authors will independently screen and review the titles and summaries. Full-texts will be considered to read if we cannot judge for inclusion based on the titles and abstracts. Any disputations regarding the study selection will be resolved by a third author through discussion. The flowchart of study selection is shown in Figure 1.

### 2.5 Data extraction and management

Two authors will independently extract data by using predefined standard data extraction form. This form includes following information: general information (first author, year of publication, location, race, sex, age, diagnostic criteria, inclusion and exclusion criteria, and funding sources); study design (sample size, details of randomization, concealment, blinding and other potential risk bias); treatments for experimental and control groups (types, dosages, frequencies, durations of treatments); and outcomes (primary and secondary outcomes, as well as adverse events). Discrepancies about data extraction will be resolved by consulting a third author.

### 2.6 Missing data dealing with

Where applicable, we will contact primary corresponding author if data are missing, insufficient, or unclear. Whenever possible, we will just analyze the available data if the missing data cannot be achieved.

### 2.7 Risk of bias assessment

The Cochrane Handbook for Systematic Reviews of Interventions Tool will be used to assess the methodological quality for included studies.^[26]^ We will judge each item of included studies according to the criteria of Cochrane risk of bias tool.^[26]^ Two authors will independently evaluate the methodological quality. Any disagreements will be settled by consensus with a third author.

### 2.8 Rating quality of evidence

We will assess the overall strength of the evidence by using Grading of Recommendations Assessment, Development, and Evaluation tool.^[27]^ The results will be presented in tables of Summary of Findings.

### 2.9 Statistical analysis

All outcome data will be pooled and will be analyzed by using RevMan 5.3 software.

#### 2.9.1 Treatment effects measurements

Continuous data are presented as mean difference with 95% confidence intervals. Standardized mean difference will be used to combine studies utilizing same outcome with different instruments.

Dichotomous data are expressed as risk ratio with 95% confidence intervals.

#### 2.9.2 Assessment of heterogeneity

Heterogeneity will be detected by using *I*^2^ test. If *I*^2^ <50, reasonable heterogeneity will be considered. Otherwise, if *I*^2^ ≥50, significant heterogeneity will be considered in this study.

#### 2.9.3 Data synthesis

If acceptable heterogeneity is identified, a fixed-effect model will be utilized to pool the data. If significant heterogeneity is found, a random-effect model will be used to pool the data. Meanwhile, subgroup analysis will also be performed. Whenever possible, meta-analysis will be conducted for the pooled data. However, if substantial heterogeneity is still found after subgroup analysis, then data will not be pooled, and meta-analysis will not be conducted. Instead, we will just report the results as a narrative summary.

#### 2.9.4 Subgroup analysis

Subgroup analysis will be conducted based on the different characteristics, intervention types, research scenario, and outcome tools.

#### 2.9.5 Sensitivity analysis

Sensitivity analysis will be carried out to check the robustness of pooled outcome data by removing low quality of studies.

#### 2.9.6 Reporting bias

If at least 10 qualified trials are included, we will apply Funnel plot and Egg regression analysis to assess the publication bias.

## 3 Discussion

O3R plays very important role for the treatment of patients with ARMD. However, up to the current, no systematic review has addressed to assess the efficacy and safety of O3R for the treatment of ARMD. The protocol of this systematic review will specifically identify the literatures on this topic emphasize interventions related to O3R for patients with ARMD. The results of this systematic review are expected to provide a summary of latest evidence on the efficacy and safety of O3R for patients with ARMD.

## Author contributions

**Conceptualization:** Yan Meng, Hong-wei Liu, Ping-ping Zhou, Jian-jie Wang.

**Data curation:** Yan Meng, Hong-wei Liu, Peng Sun, Ping-ping Zhou, Jian-jie Wang.

**Formal analysis:** Yan Meng, Hong-wei Liu, Peng Sun.

**Funding acquisition:** Hong-wei Liu.

**Investigation:** Jian-jie Wang.

**Methodology:** Yan Meng, Hong-wei Liu, Peng Sun, Ping-ping Zhou.

**Project administration:** Jian-jie Wang.

**Resources:** Yan Meng, Hong-wei Liu, Peng Sun, Ping-ping Zhou.

**Software:** Yan Meng, Hong-wei Liu, Peng Sun, Ping-ping Zhou.

**Supervision:** Jian-jie Wang.

**Validation:** Yan Meng, Hong-wei Liu, Peng Sun, Jian-jie Wang.

**Visualization:** Yan Meng, Peng Sun, Ping-ping Zhou, Jian-jie Wang.

**Writing – original draft:** Yan Meng, Hong-wei Liu, Peng Sun, Ping-ping Zhou, Jian-jie Wang.

**Writing – review & editing:** Yan Meng, Hong-wei Liu, Ping-ping Zhou, Jian-jie Wang.
